# From Bench to Bedside: New Frontiers in Understanding and Treating Postoperative Delirium

**DOI:** 10.3390/jcm14238418

**Published:** 2025-11-27

**Authors:** Kenneth Meza Monge, Allison L. B. Shapiro, Christina Coughlan, Benedetto Mungo, Richard Schulick, Akshay Pratap, Elizabeth J. Kovacs, Juan-Pablo Idrovo

**Affiliations:** 1 Division of G.I., Trauma, and Endocrine Surgery, Department of Surgery, Anschutz Medical Campus, University of Colorado, Aurora, CO 80045, USA; akshay.chauhan@cuanschutz.edu (A.P.); elizabeth.kovacs@cuanschutz.edu (E.J.K.); juan.idrovo@cuanschutz.edu (J.-P.I.); 2 Department of Pediatrics, Section of Endocrinology, School of Medicine, Anschutz Medical Campus, University of Colorado, Aurora, CO 80045, USA; allison.shapiro@cuanschutz.edu; 3 Lifecourse Epidemiology of Adiposity and Diabetes Center, Anschutz Medical Campus, University of Colorado, Aurora, CO 80045, USA; 4 Department of Neurology, School of Medicine, Anschutz Medical Campus, University of Colorado, Aurora, CO 80045, USA; christina.coughlan@cuanschutz.edu; 5 CU Alzheimer’s and Cognition Center (CUACC), Anschutz Medical Campus, University of Colorado, Aurora, CO 80045, USA; 6 Division of Surgical Oncology, Department of Surgery, Anschutz Medical Campus, University of Colorado, Aurora, CO 80045, USA; benedetto.mungo@cuanschutz.edu (B.M.); richard.schulick@cuanschutz.edu (R.S.); 7 Cancer Center, Anschutz Medical Campus, University of Colorado, Aurora, CO 80045, USA; 8 Burn and Alcohol Research Program, Department of Surgery, Anschutz Medical Campus, University of Colorado, Aurora, CO 80045, USA

**Keywords:** postoperative delirium, neuroinflammation, blood–brain barrier dysfunction, cholinergic signaling, aged rodent models, translational research

## Abstract

Postoperative delirium is a frequent and serious neurocognitive complication in older surgical patients, characterized by acute impairments in attention, awareness, and cognition. It is associated with increased morbidity, prolonged hospitalization, and persistent cognitive decline. In this narrative review, we synthesize translational research on biological mechanisms underlying delirium and emerging targeted interventions. We conducted a comprehensive search of major biomedical databases, with no date restrictions but prioritizing publications from 2018 to 2025. The multifactorial pathophysiology involves dysregulated cholinergic and dopaminergic signaling, systemic and neuroinflammation, oxidative stress, and metabolic disturbances. Pre-existing cognitive impairment and frailty emerge as key clinical risk factors linked to these mechanisms. Aged rodent models replicate delirium-like cognitive deficits and validate mechanistic pathways, while human neuroimaging studies demonstrate disrupted functional connectivity in attentional and consciousness networks. Genomic and proteomic analyses have identified candidate biomarkers for early detection and risk stratification, and genetic variants associated with inflammation and neurodegeneration contribute to individual vulnerability. Emerging therapies targeting inflammation, microglial activation, mitochondrial function, and neurotransmitter balance show promise in preclinical studies, although clinical trials report mixed results. We advocate integrating basic science with clinical care through thorough preoperative assessment, multicomponent non-pharmacological strategies, and mechanism-based preventive measures to reduce the burden of postoperative delirium.

## 1. Introduction

Postoperative delirium represents one of the most common and consequential complications following surgery, particularly among older adults. This acute neuropsychiatric syndrome is characterized by fluctuating disturbances in attention, awareness, and cognition that typically develop over a short period following surgical intervention [1,2]. The incidence varies considerably depending on patient demographics, surgical type, and assessment methods, ranging from 10–15% in general surgery to 30–65% in high-risk populations, such as those undergoing emergency orthopedic trauma procedures, particularly hip fracture repair, or cardiac surgery [3,4]. Evidence consistently demonstrates that emergency surgery carries a substantially higher delirium risk than elective procedures, with odds ratios ranging from 2.8 to 5.6 across multiple studies [5].

The clinical significance of postoperative delirium extends far beyond its acute presentation. This syndrome is associated with increased mortality, prolonged hospitalization, higher rates of institutional care placement, long-term cognitive decline, and substantial healthcare costs [6,7]. Recent studies have established that postoperative delirium often serves as a trigger for long-term cognitive impairment and even dementia, with a meta-analysis showing a two-fold increased risk of incident dementia among patients experiencing delirium [8]. Recent economic analyses estimate that delirium contributes to more than $182 billion in annual healthcare expenditures in the United States alone, with a disproportionate burden falling on older adult populations [9]. Despite its prevalence and impact, postoperative delirium remains underdiagnosed, undertreated, and incompletely understood at a mechanistic level [10].

This review synthesizes the current landscape of translational research in postoperative delirium, examining how advances in neurobiology, neuroinflammation, genetics, and novel research methodologies collectively enhance our understanding of delirium pathophysiology and inform innovative therapeutic approaches [11,12]. Both the acute presentation of delirium and its potential progression to long-term cognitive impairment will be addressed throughout this review, paying particular attention to the mechanisms that possibly drive the transition from transient to persistent neurocognitive dysfunction. Historical approaches to delirium management have been largely reactive and symptomatic, primarily focused on introducing environmental modifications, administering antipsychotic medications, and providing supportive care [13]. These interventions often fail to address the underlying pathophysiology and have shown limited efficacy in preventing or treating postoperative delirium [14]. Contemporary research offers valuable insights into the biological mechanisms of delirium that can serve as targets for prevention, early detection, and mechanism-based interventions [9]. Understanding these mechanisms is essential for developing effective clinical strategies that move beyond symptom management to address the underlying causes of this complex syndrome.

## 2. Materials and Methods

We conducted a comprehensive literature search using PubMed, Scopus, EMBASE, PsycINFO, and CENTRAL databases on 12 February 2025, with an update on 15 March 2025, to identify relevant publications addressing the pathophysiology, biomarkers, animal models, and emerging therapeutic strategies for postoperative delirium. Our search strategy combined concept blocks related to delirium (delirium OR “acute brain dysfunction” OR “acute brain failure” OR “acute cognitive change”) AND postoperative settings (postoperative OR perioperative OR surgery OR surgical) with targeted mechanistic terms (neuroinflammation OR “blood-brain barrier” OR cholinergic OR microglia OR “rodent models” OR APOE OR biomarkers OR “omics technologies”) using appropriate Boolean operators and subject headings for each database. We also searched trial registries (ClinicalTrials.gov and WHO ICTRP) to identify ongoing studies. No date restrictions were applied to maximize the breadth of included literature, though we prioritized contemporary research (2018–2025) while including foundational studies.

Two reviewers independently screened abstracts and titles for relevance, with conflicts resolved by a third reviewer. Reference lists of selected articles and relevant reviews were manually searched to identify additional sources. We included primary research studies (randomized controlled trials, cohort studies, case–control studies), high-quality systematic reviews and meta-analyses, and key mechanistic studies in animal models that provided insights into delirium pathophysiology or treatment. We excluded case reports, editorials, and non-English language publications without available translations.

The quality of included studies was evaluated using the appropriate assessment tools (GRADE for intervention studies, QUADAS-2 for diagnostic accuracy studies, and SYRCLE’s risk of bias tool for animal studies). A total of 412 articles were reviewed in full text, with 212 ultimately included in the final narrative synthesis based on their scientific rigor, relevance, and contribution to the thematic domains of this review. While this review was not pre-registered, we followed PRISMA guidelines for the search and selection process. To improve clarity and grammar, we used Grammarly, a language-editing software, for minor proofreading during manuscript preparation. No generative artificial intelligence (AI) tools were used to create, analyze, or interpret scientific content. All text, analyses, and conclusions were fully developed and verified by the authors.

## 3. Fundamental Neurobiology of Delirium

### 3.1. Neuroanatomical Substrates of Attention and Consciousness

Delirium fundamentally represents a disorder of attention and consciousness, functions that depend on the integrated activity of distributed neural networks [15,16]. The ascending reticular activating system (ARAS), originating in the brainstem and projecting to the thalamus and cerebral cortex, plays a critical role in arousal and consciousness [17]. Disruption of this pathway has been directly linked to delirium symptoms in both preclinical models (primarily in aged rodents) and human clinical studies [18].

Recent advances in network neuroscience have enhanced our understanding of consciousness beyond the classical ARAS model. Contemporary theories, such as the Global Neuronal Workspace Hypothesis and Integrated Information Theory, propose that consciousness emerges from the dynamic integration of information across specialized brain regions [19]. These frameworks help explain how delirium may result from temporary disconnection between brain networks rather than focal damage, consistent with its fluctuating nature and potential for complete recovery.

Attention networks, comprising anterior cingulate, prefrontal, and parietal cortical regions, show altered connectivity and activity during episodes of delirium in human patients [20]. Advanced neuroimaging techniques have revealed reduced functional connectivity within the default mode network and between frontoparietal regions in patients experiencing delirium [21,22]. These findings align with the clinical presentation of impaired attention, disorganized thinking, and altered consciousness that characterize the syndrome.

### 3.2. Key Neurotransmitter Systems in Delirium

Multiple neurotransmitter systems become dysregulated during postoperative delirium, with strong evidence implicating acetylcholine, dopamine, gamma-aminobutyric acid (GABA), and glutamate [23,24] (Table 1). The neurotransmitter hypothesis of delirium posits that an imbalance in these systems, either absolute or relative to each other, underlies the diverse clinical manifestations of the syndrome.

Cholinergic dysfunction represents one of the most extensively documented neurochemical abnormalities in delirium [25]. Acetylcholine plays crucial roles in attention, memory, and arousal; thus, it makes sense that serum anticholinergic activity has been shown to correlate with the severity of delirium in both surgical and medical patients with correlation coefficients ranging from 0.45 to 0.63 (*p* < 0.01) in prospective human studies [26]. Preclinical models utilizing anticholinergic drugs (such as scopolamine and atropine) in rodents (primarily mice and rats) demonstrate that cholinergic antagonists induce delirium-like behavioral changes within hours of administration, while cholinergic agonists can attenuate these effects when administered either prophylactically or therapeutically [27,28].

Despite the strong biological rationale, clinical trials testing cholinesterase inhibitors for delirium prevention and treatment have yielded disappointing results. A landmark multicenter trial by van Eijk et al. [29] found that rivastigmine not only failed to reduce delirium duration but was associated with increased mortality, leading to early trial termination. This discrepancy between preclinical promise and clinical disappointment highlights the challenges in translating mechanistic findings to effective interventions, likely reflecting the complex, multifactorial nature of delirium and potential differences between animal models and human disease.

The reciprocal relationship between cholinergic and dopaminergic activity explains why dopaminergic excess, particularly in relation to cholinergic deficiency, contributes significantly to the pathophysiology of delirium [30]. This provides a mechanistic rationale for the therapeutic efficacy of antipsychotic medications, which primarily act through dopamine receptor antagonism [31]. However, it is important to note that while antipsychotics have a sound biological basis, systematic reviews and clinical practice guidelines have not consistently recommended their routine use for delirium prevention due to limited efficacy evidence and potential adverse effects, particularly in older adults [32,33]. The dopaminergic system comprises five distinct receptor subtypes (D1–D5), with D2 receptors, expressed throughout corticostriatal circuits, particularly relevant to delirium pathophysiology and treatment [34]. D2 receptors are involved in attention and executive function, and their blockade forms the basis for most antipsychotic medications used in delirium management. Animal models (primarily in rats aged 18–24 months) demonstrate that dopaminergic agonists induce hyperactive delirium-like behaviors within 1–2 h of administration, while selective D2 receptor antagonists can ameliorate these symptoms, observed as dose-dependent reductions in locomotor activity by 45–60% and improvement in attention-based tasks such as the 5-choice serial reaction time test [35].

Human cerebrospinal fluid (CSF) studies provide direct evidence of neurotransmitter dysfunction in POD. CSF acetylcholine levels decrease by 35–50% during delirium episodes compared to non-delirious controls, with the magnitude of reduction correlating with delirium severity. Conversely, CSF dopamine metabolites (homovanillic acid, HVA) increase by 40–70% in hyperactive delirium, supporting the dopaminergic excess hypothesis. The ratio of HVA to 5-hydroxyindoleacetic acid (5-HIAA, serotonin metabolite) predicts delirium phenotype, with ratios > 2.5 associated with hyperactive presentations. Neuroimaging studies using [11C] donepezil PET demonstrate reduced cholinergic activity in frontoparietal networks during POD, while [123I]FP-CIT SPECT reveals altered dopamine transporter availability in striatal regions [36]. These findings directly link preclinical mechanistic insights to human pathophysiology, validating neurotransmitter-targeted therapeutic approaches.

### 3.3. Blood–Brain Barrier Integrity and Neuronal Energetics

The blood–brain barrier (BBB) (neurovascular unit) is a critical interface between the peripheral circulation and central nervous system [37], with clinical and preclinical evidence indicating that BBB disruption represents a key mechanism in the pathophysiology of postoperative delirium [38,39]. Surgical trauma induces systemic inflammation that compromises the integrity of the BBB through multiple mechanisms, including activation of endothelial cells, alterations in localization of tight junction proteins, disruption of astrocyte end feet that form part of the neurovascular unit, and increased paracellular permeability [40]. Human studies have shown that plasma biomarkers of BBB disruption, such as S100β, correlate with the incidence of delirium in postoperative patients, with measurements typically obtained 24–48 h after surgery [41].

In addition, disruption of energy metabolism resulting from aberrant mitochondrial function represents an emerging mechanism in delirium pathophysiology [42,43,44,45]. Surgical stress induces oxidative stress, inflammatory damage to mitochondria, and altered cerebral glucose metabolism, collectively impairing neuronal energetics [46]. Positron emission tomography studies in human patients have shown reduced cerebral glucose metabolism during delirium episodes by 15–30%, particularly in frontoparietal regions associated with attention and executive function [47]. These insults and the brain’s high-energy demand make it particularly vulnerable to injury [44].

Having established the fundamental neurobiology of POD, we now examine the central role of neuroinflammation and blood–brain barrier dysfunction, which represent key therapeutic targets for clinical intervention.

## 4. Neuroinflammation and Stress Responses

The temporal and spatial dynamics of POD pathophysiology unfold in overlapping phases that interact synergistically. Within 2–6 h of surgical incision, peripheral inflammatory mediators (IL-6, TNF-α, IL-1β) surge 2–10 fold above baseline [48]. This peripheral inflammatory response triggers blood–brain barrier disruption within 6–12 h, evidenced by increased CSF/serum albumin ratios and MRI-detected gadolinium enhancement [49]. Microglial activation in hippocampal and prefrontal regions peaks at 24–48 h postoperatively, coinciding with maximal neuroinflammatory marker elevation (GFAP, S100β) [50]. Neurotransmitter dysfunction begins immediately with anesthetic exposure but is amplified by inflammatory processes over 24–72 h, with CSF acetylcholine decreasing by 35–50% and dopamine metabolites increasing by 40–70% [51]. Metabolic derangements develop progressively, with mitochondrial dysfunction detectable by 12 h (reduced ATP production, increased reactive oxygen species) and oxidative stress markers (malondialdehyde, 8-isoprostane) peaking at 24–48 h [52]. These processes interact bidirectionally: neuroinflammation enhances cholinergic dysfunction through α7 nicotinic receptor downregulation, oxidative stress amplifies microglial activation via NF-κB pathways, and metabolic disturbances exacerbate neurotransmitter imbalances through impaired synthesis and reuptake mechanisms. This temporal cascade explains the typical POD onset at 24–72 h and supports early intervention strategies targeting these mechanisms.

### 4.1. Central and Peripheral Inflammatory Mechanisms

**Figure 1:**
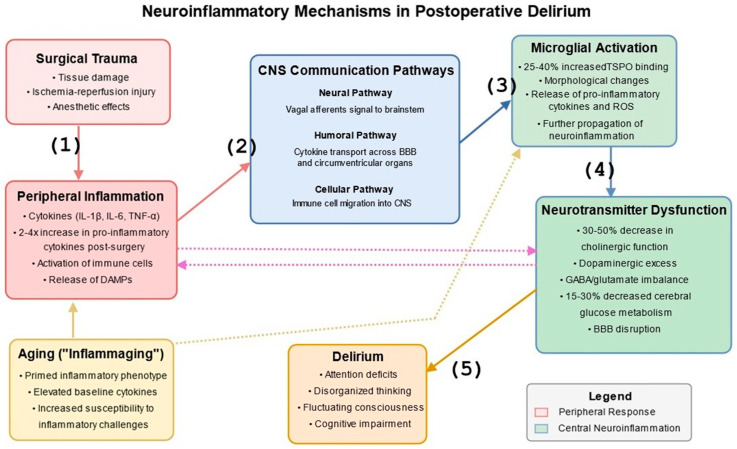
Neuroinflammatory Mechanisms in Postoperative Delirium. This figure illustrates the bidirectional communication between peripheral inflammatory responses to surgical trauma and central neuroinflammatory processes. The diagram shows: (1) Surgical trauma inducing systemic inflammation with release of pro-inflammatory cytokines, (2) Three pathways of peripheral-to-central inflammatory communication (neural via vagus nerve, humoral via BBB transporters, and cellular via immune cell migration), (3) Microglial activation in the brain leading to local cytokine release, (4) Effects on neurotransmitter systems, particularly acetylcholine and dopamine, (5) Resulting neural network dysfunction and clinical manifestations of delirium. The figure also highlights the critical role of aging (“inflammaging”), shown at the bottom, with its primed inflammatory phenotype, elevated baseline cytokines, and increased susceptibility to inflammatory challenges, creating vulnerability to peripheral inflammation and central microglial activation. Bidirectional arrows between peripheral and central compartments indicate the complex feedback loops that can amplify and sustain the inflammatory cascade. Quantitative relationships are marked by showing approximately 2–4-fold increases in peripheral cytokines following surgery, 25–40% increased microglial activation (measured via TSPO binding), and 30–50% decreases in cholinergic function during delirium episodes. References: [42,43,44,46,47,48,49,50,51,52].

Postoperative delirium emerges at the intersection of peripheral inflammatory responses to surgical trauma, anesthesia, and central neuroinflammatory processes [42,43,44,46,47,48,49,50,51,52] (Figure 1). Surgical procedures trigger a robust peripheral immune response characterized by the release of pro-inflammatory cytokines, damage-associated molecular patterns (DAMPs), and activation of innate immune cells [53]. This systemic inflammatory response initiates a cascade of events that ultimately affect brain function through multiple pathways [51].

Communication between peripheral inflammation and the central nervous system (CNS) occurs through several complementary routes [54]. The neural pathway involves afferent vagal nerve signaling, which directly transmits inflammatory information from the periphery to brainstem nuclei and higher brain structures [55]. The humoral pathway encompasses the transport of circulating cytokines across the BBB through specific transport proteins, as well as diffusion at specialized regions lacking a conventional BBB known as circumventricular organs (including the area postrema, median eminence, subfornical organ, and organum vasculosum of the lamina terminalis). The cellular pathway involves activated peripheral immune cells that migrate into the CNS, particularly under conditions of BBB disruption [56].

“Inflamm-aging” is a chronic, low-grade inflammatory state that develops with advancing age and creates a pro-inflammatory neural environment that responds more vigorously to peripheral inflammatory challenges [48,57,58]. The brain in older subjects exhibits a primed inflammatory phenotype characterized by activated microglia and elevated baseline cytokine levels, which may explain the increased vulnerability of older patients to postoperative delirium [49,58].

### 4.2. Cytokine and Chemokine Signaling and Microglial Activation

Cytokines and chemokines are key mediators in the neuroinflammatory cascade associated with postoperative delirium [58]. Elevated levels of pro-inflammatory cytokines, including IL-1β, IL-6, and TNF-α, have been consistently documented in the serum and cerebrospinal fluid of patients experiencing delirium, with meta-analyses showing 2–4-fold increases compared to non-delirious patients [48]. Additionally, chemokines such as CXCL8 (formerly known as IL-8), CCL2, and CXCL10 play essential roles in recruiting and directing immune cells to sites of inflammation and are elevated in the circulation of delirious patients by 2–3-fold compared to non-delirious controls. These inflammatory mediators directly affect neuronal function through multiple mechanisms, including modulation of neurotransmitter release, alteration of synaptic plasticity, and disruption of neuronal network synchronization, which collectively impair cognition and attention [49].

IL-1β has emerged as an essential mediator in delirium pathophysiology [50]. This cytokine is produced primarily by activated microglia and infiltrating monocytes/macrophages in the brain, as well as by peripheral immune cells that release it into circulation. In the CNS, IL-1β acts on neurons, astrocytes, and oligodendrocytes through the IL-1 receptor type 1. This cytokine disrupts long-term potentiation, impairs synaptic plasticity, and reduces cholinergic transmission—effects that align with the cognitive symptoms of delirium [51]. Preclinical models in aged mice (18–24 months) demonstrate that IL-1 receptor antagonists can prevent surgery-induced cognitive dysfunction and delirium-like behaviors when administered before or shortly after surgery, reducing memory deficits by 65–80% in standardized behavioral tests such as the Morris water maze and novel object recognition test measured 1–3 days post-surgery [50].

This mechanistic evidence has spurred clinical investigations of anti-inflammatory agents for delirium prevention. Recent trials by Clemmensen et al. [59] and Kluger et al. [60] investigated high-dose perioperative glucocorticoids in hip fracture patients, showing promising reductions in delirium incidence (relative risk reductions of 25–35%). These findings provide translational validation of the inflammatory hypothesis of delirium and suggest potential for targeted anti-inflammatory strategies in high-risk surgical populations.

Gut-derived bacterial products, particularly lipopolysaccharide (LPS) and other endotoxins, can contribute to neuroinflammation in postoperative delirium. Surgery and anesthesia can compromise intestinal barrier integrity, allowing bacterial translocation and the release of endotoxins into circulation. These molecules can activate peripheral immune cells and directly stimulate innate immune receptors in the brain, particularly Toll-like receptor 4 (TLR4), further propagating neuroinflammatory responses [61].

Among the best-studied resident immune cells of the brain, microglia can be activated in response to direct CNS insults and peripheral inflammatory signals [62]. Upon activation, microglia undergo morphological changes, proliferate, and release pro-inflammatory cytokines, chemokines, and reactive oxygen species that further propagate neuroinflammation [63]. Advanced imaging techniques using PET with translocator protein (TSPO) ligands have visualized microglial activation in human patients experiencing delirium, with 25–40% increased binding potential in hippocampal and prefrontal regions, which underly memory and executive processes, compared to non-delirious controls, typically measured 3–7 days after surgery [64]. 

### 4.3. Surgical Stress Response and Oxidative Stress

Surgical intervention triggers a coordinated neuroendocrine stress response that significantly impacts brain function and behavior through the hypothalamic–pituitary–adrenal (HPA) axis. Cortisol modulates inflammatory processes, neurotransmitter systems, and neural excitability. Multiple human studies have implicated dysregulated HPA axis activity in delirium pathophysiology [65].

Hyperactivation of the HPA axis following surgery results in sustained cortisol elevation, which exerts complex effects on brain function [66]. While acute cortisol increases may initially serve adaptive functions, prolonged elevation disrupts neural networks, damages vulnerable neurons, particularly in the hippocampus, and impairs cognitive processes [67]. Clinical studies have demonstrated that higher postoperative cortisol levels (measured 24–72 h after surgery) correlate with delirium incidence and severity across multiple surgical populations, particularly in older patients undergoing major orthopedic or cardiac procedures [68].

Oxidative stress represents a critical mechanism linking surgical trauma, inflammation, and neuronal dysfunction in delirium pathophysiology [69]. Surgical procedures induce oxidative stress through tissue injury, ischemia–reperfusion events, and inflammatory cell activation [70]. Studies in both animal models (aged rats) and clinical populations demonstrate increased oxidative stress markers during episodes of delirium, with 3–5-fold elevations in plasma malondialdehyde levels and 4–6-fold increases in hippocampal malondialdehyde (measured 24–48 h post-surgery), along with reductions in brain and plasma antioxidant capacity by 30–40% [71].

### 4.4. Animal Models Validating Inflammatory Mechanisms

Animal models have provided crucial insights into the inflammatory mechanisms underlying postoperative delirium [72,73] (Table 2). These models recapitulate key features of clinical delirium while allowing for detailed investigation of molecular pathways, causal relationships, and potential therapeutic interventions. Multiple complementary rodent models have been developed to investigate different aspects of postoperative delirium, each offering distinct advantages for translational research [51,69].

The tibia fracture model in mice represents one of the most well-characterized preclinical approaches to studying postoperative delirium [70]. In aged mice (18–24 months), tibia fracture under general anesthesia (typically isoflurane for 20–30 min) induces cognitive deficits, attention impairments, and altered arousal that parallel clinical delirium when assessed 1–3 days after surgery [79]. This model demonstrates marked neuroinflammation, with microglial activation and elevated pro-inflammatory cytokine protein levels in the hippocampus and prefrontal cortex [76]. Importantly, young adult mice (3–4 months) subjected to the same procedure show significantly less cognitive impairment and neuroinflammation, highlighting the importance of advanced age as a key risk factor for delirium.

The abdominal laparotomy model is another clinically relevant paradigm that closely mimics human surgical experiences [77]. Studies have demonstrated that laparotomy in aged mice (18–24 months) under isoflurane anesthesia (30–45 min) induces significant hippocampal neuroinflammation and cognitive impairments that persist for 3–7 days postoperatively, with peak deficits typically observed 24–72 h after surgery [78]. The laparotomy model is particularly valuable for its ability to recapitulate the dynamic temporal profile of human postoperative delirium, with peak cognitive dysfunction occurring 24–72 h after surgery, followed by gradual resolution in most animals [80]. Importantly, this model demonstrates age-dependent vulnerability, with aged mice (18–24 months) showing more pronounced and persistent cognitive deficits compared to young adult mice (3–6 months), mirroring the increased susceptibility of elderly patients to postoperative delirium [81].

A critical question in delirium research is how well these animal models map to human delirium phenotypes, particularly hypoactive delirium, which is associated with worse clinical outcomes. Most rodent models primarily capture features analogous to hyperactive delirium (increased locomotor activity, agitation) rather than hypoactive presentations. Recent work by Vasunilashorn and colleagues [82] has begun to develop and validate models of hypoactive-like behavior in aged rodents by measuring reduced exploration, decreased motor activity, and diminished responsiveness to environmental stimuli. This represents an important advance, though the correspondence between animal behaviors and human clinical phenotypes requires careful interpretation and validation.

Researchers employ standardized cognitive and behavioral assessments to quantify delirium-like behaviors in animal models (Table 3). The open field test comprehensively evaluates locomotor activity, anxiety-like behavior, and exploration tendencies [71,83]. Aged mice (18–24 months) subjected to surgical procedures typically exhibit reduced center exploration (by 30–50%), decreased total distance traveled (by 40–60%), and increased corner preference when tested 24–48 h post-surgery, indicative of anxiety-like states that parallel features of clinical delirium [71]. The novel object recognition test assesses recognition memory by measuring preferential exploration of novel objects with impaired discrimination between familiar and novel objects. A reduced discrimination index (40–70% from baseline) has been measured in animals tested 1–3 days post-surgery, reflecting cognitive dysfunction consistent with delirium [84]. The Y-maze spontaneous alternation test evaluates working memory by measuring sequential entry patterns into maze arms. A reduction in the alternation percentages from baseline 60–75% to 30–40% was observed 24–72 h post-surgery, implying cognitive impairment [85]. Collectively, these behavioral tests model different aspects of the complex clinical presentation of delirium, providing a multidimensional profile of cognitive and neuropsychiatric alterations [71].

Biomarker analysis in these models has identified several key indicators of neuroinflammation that correlate with cognitive impairment (Table 4). Pro-inflammatory cytokine proteins, particularly IL-1β, IL-6, and TNF-α, show significant upregulation in both the periphery (2–5-fold increases in plasma) and central nervous system (3–10-fold increases in hippocampus) following surgery in aged mice (18–24 months), with peak levels occurring 24–72 h post-surgery and corresponding to periods of maximal cognitive dysfunction [86]. Microglial activation markers, including Iba1 and CD68, demonstrate increased protein expression in the hippocampus (40–120% increase in immunoreactivity) and prefrontal cortex 1–3 days after surgery, reflecting activation of resident immune cells in brain regions critical for cognitive function [87]. Disruption of the BBB can be quantified through increased permeability to circulating dyes or proteins, with 50–200% increases in extravasation rates 24–48 h following surgery in aged rodents [88]. For example, markers of oxidative stress measured 24–72 h post-surgery, including malondialdehyde (increased by 30–90% in brain tissue) and reduced glutathione (decreased by 20–40% in hippocampus), further display the neuroinflammatory environment in these rodent models [89].

The high-mobility group box 1 protein (HMGB1) is a DAMP released from injured tissues following surgical trauma, which triggers innate immune responses through receptors including TLR4 and RAGE. HMGB1 has emerged as a particularly important mediator and biomarker in surgery-induced neuroinflammation [90]. Neutralization through antibody administration or receptor antagonism, when administered either prophylactically or up to 6 h after the tibia fracture or laparotomy models of surgery in aged mice, significantly attenuating postoperative neuroinflammation and cognitive dysfunction, reducing microglial activation 50–70% and improving cognitive performance by 60–80% when assessed/measured/determined using behavioral tests conducted 1–7 days post-surgery [91].

Intervention studies using these rodent models have validated the causal role of inflammation in delirium-like phenotypes [92]. Anti-inflammatory approaches, including cytokine antagonists (particularly IL-1 receptor antagonists), microglial modulators (compounds that reduce microglial pro-inflammatory activation without depleting the cells, such as minocycline), and targeted anti-inflammatory compounds, when assessed 1–7 days after surgery, consistently attenuate postoperative cognitive dysfunction across multiple rodent model systems, with improvements in cognitive performance of 60–85% compared to untreated controls, depending on the specific intervention and outcome measure [93,94]. The complementary nature of different animal models enhances translational relevance by allowing researchers to identify conserved mechanisms across diverse surgical contexts, ultimately facilitating the development of targeted preventive and therapeutic approaches for postoperative delirium [95].

## 5. Pre-Existing Cognitive Impairment and Frailty as Risk Factors

Pre-existing cognitive impairment represents the most consistently documented risk factor for postoperative delirium, with multiple systematic reviews establishing a 2–5 fold increased risk compared to cognitively intact individuals [96]. This relationship reflects a “cognitive reserve” model, where underlying pathology reduces the brain’s resilience to perioperative stressors. Importantly, even mild cognitive impairment that may be undetected without formal assessment significantly increases delirium risk, highlighting the importance of preoperative cognitive screening [97].

The biological mechanisms underlying this vulnerability include pre-existing neuroinflammation, reduced cholinergic function, and compromised neural network integrity. Neuropathological studies have demonstrated that individuals with Alzheimer’s disease pathology (even preclinical) show exaggerated neuroinflammatory responses to peripheral inflammatory stimuli, with microglial priming creating a sensitized state [98]. This aligns with the clinical observation that postoperative delirium often unmasks underlying neurodegenerative processes rather than creating them de novo.

Frailty has emerged as another potent risk factor for delirium, potentially more predictive than chronological age alone. Defined as a state of increased vulnerability to stressors resulting from diminished physiological reserves across multiple systems, frailty is associated with a 2–3-fold increased delirium risk [99]. The biological underpinnings of this association include elevated baseline inflammation, impaired stress response systems, and reduced physiological reserve that limits the ability to maintain homeostasis during perioperative stress.

Comprehensive geriatric assessment (CGA) approaches that address these risk factors have shown efficacy in reducing postoperative delirium. The landmark study by Partridge et al. [100] demonstrated that preoperative CGA with targeted interventions reduced postoperative delirium incidence by 40% in high-risk surgical patients. This approach connects clinical risk factor identification with biological mechanisms by addressing modifiable contributors to inflammation, stress responses, and metabolic derangements before surgical insult occurs.

## 6. Genetic Factors and Biomarker Development

### Genetic Predisposition and Risk Alleles

**Figure 2:**
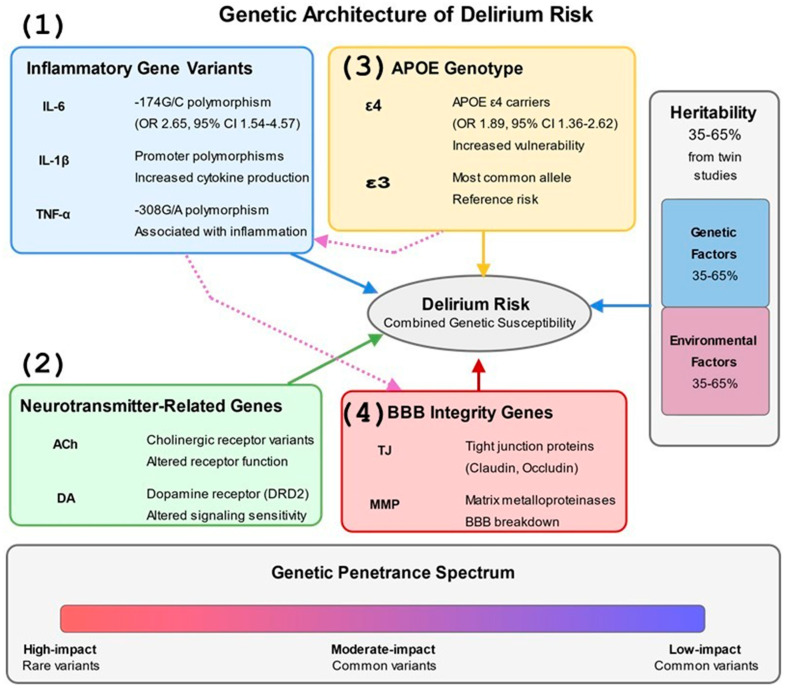
Genetic Architecture of Delirium Risk. This figure depicts the complex genetic architecture underlying delirium susceptibility in humans. The diagram presents four major genetic influence categories: (1) Inflammatory gene variants (IL-6, IL-1β, TNF-α) with specific polymorphisms and their associated odds ratios, including the IL-6 -174G/C polymorphism (OR 2.65, 95% CI 1.54–4.57), (2) Neurotransmitter-related genes affecting cholinergic and dopaminergic signaling, (3) APOE genotype, with ε4 carriers showing increased vulnerability (OR 1.89, 95% CI 1.36–2.62) compared to the common ε3 allele, and (4) Blood–brain barrier integrity genes including tight junction proteins and matrix metalloproteinases. The figure illustrates the relative contribution of genetic factors (35–65% based on twin studies) versus environmental factors to delirium risk in the vertical bar chart on the right. The bottom section shows the genetic penetrance spectrum from high-impact rare variants to common low-impact polymorphisms. Dotted arrows between gene categories indicate cross-pathway interactions, such as APOE’s influence on inflammatory responses and inflammatory genes’ effects on BBB integrity, highlighting the interconnected nature of genetic risk factors. References: [88,89,90,91,92,93,94,95,96,97].

Emerging evidence from human studies, based on twin and family studies, supports a substantial genetic component to delirium susceptibility, with heritability estimates ranging from 35 to 65% [101]. Genetic variation influences delirium risk through multiple mechanisms, including effects on inflammatory responses, neurotransmitter function, blood–brain barrier integrity, and stress reactivity [102] (Figure 2). Genome-wide and candidate gene studies in humans have identified several polymorphisms associated with increased delirium vulnerability across surgical populations [103].

Specific genetic polymorphisms demonstrate robust associations with POD vulnerability. The APOE ε4 allele, present in approximately 25% of the population, confers a 1.9-fold increased risk through mechanisms involving enhanced neuroinflammation, impaired amyloid clearance, and reduced synaptic plasticity. The IL-6 -174G/C polymorphism influences cytokine transcription, with the high-producing G allele associated with 2.65-fold increased POD risk (95% CI 1.54–4.57) in cardiac surgery patients. Additional variants in TNF-α (rs1800629), IL-1β (rs16944), and CXCL8 (rs4073) modify inflammatory responses to surgical stress. The COMT Val158Met polymorphism affects dopamine metabolism, with the Met allele associated with reduced enzymatic activity and altered delirium phenotypes. Matrix metalloproteinase polymorphisms (MMP-9 rs3918242) influence blood–brain barrier integrity, while variants in tight junction proteins (CLDN5, OCLN) modulate barrier permeability during systemic inflammation.

Genes regulating inflammatory pathways strongly correlate with postoperative delirium risk in human patients [104]. Polymorphisms in cytokine genes, including IL-6, CXCL8, and TNF-α, modify baseline inflammatory status and the magnitude of inflammatory responses to surgical stress [105]. For example, the -174G/C polymorphism in the IL-6 promoter region influences transcriptional activity and cytokine production, with the high-producing G allele conferring increased delirium risk following cardiac surgery in older patients (odds ratio 2.65, 95% CI 1.54–4.57) [106]. The apolipoprotein E (APOE) gene, particularly the ε4 allele associated with Alzheimer’s Disease risk, has been extensively studied in postoperative delirium [107]. Meta-analyses indicate that APOE ε4 carriers exhibit approximately twice the risk of developing delirium in patients following major surgery, particularly orthopedic and cardiac procedures, compared to non-carriers (pooled OR 1.89, 95% CI 1.36–2.62) [108]. However, recent research suggests this association may be more complex and context-dependent than previously thought, with interactions between APOE genotype and inflammatory markers like C-reactive protein potentially modifying risk profiles [109]. It should be noted that some studies have failed to replicate this association in specific surgical populations, and the effect appears to be modulated by factors such as age, surgical type, and concurrent pathologies [110].

## 7. Epigenetic Modifications and Biomarkers

Beyond genetic variation, dynamic epigenetic modifications are an essential mechanism linking surgical stress to altered gene expression and the pathophysiology of delirium [109,111]. Epigenetic processes, including DNA methylation, histone modifications, and non-coding RNA regulation, respond to environmental stimuli and modify gene transcription without altering the underlying DNA sequence [112]. In human patients, surgery induces significant epigenetic reprogramming in both peripheral immune cells and CNS tissues, with changes in DNA methylation at inflammatory gene promoters ranging from 10 to 35% when measured 24–72 h after surgery [113,114].

Biomarkers represent measurable indicators of normal biological processes, pathogenic mechanisms, or pharmacologic responses that can inform clinical decision-making [115]. For postoperative delirium in human patients, biomarker development has focused primarily on inflammatory mediators, indicators of neuronal injury, and metabolic factors reflecting cerebral energetics [116] (Table 5). These biomarkers serve multiple functions, including preoperative risk stratification, early detection, clinical course monitoring, and intervention efficacy assessment [117].

Inflammatory biomarkers, including cytokines, chemokines, and acute-phase proteins, correlate with delirium incidence and severity in human patients [118]. Preoperative elevation of IL-6 (OR 2.32, 95% CI 1.54–3.49), CXCL8 (OR 3.05, 95% CI 1.86–4.99), and C-reactive protein (OR 2.13, 95% CI 1.60–2.83) independently predict delirium risk across surgical populations, suggesting baseline inflammatory status as a modifiable risk factor [48].

Neuronal injury biomarkers reflect CNS damage and dysfunction during delirium [119,120,121]. Neuron-specific enolase (NSE), S100β protein, glial fibrillary acidic protein (GFAP), ubiquitin carboxy-terminal hydrolase L1 (UCHL-1), and neurofilament light chain (NFL) increase significantly in the serum and cerebrospinal fluid obtained from patients experiencing delirium 24–72 h post-surgery, with average increases of 40–120% above baseline levels compared to non-delirious surgical patients [122,123].

**Figure 3:**
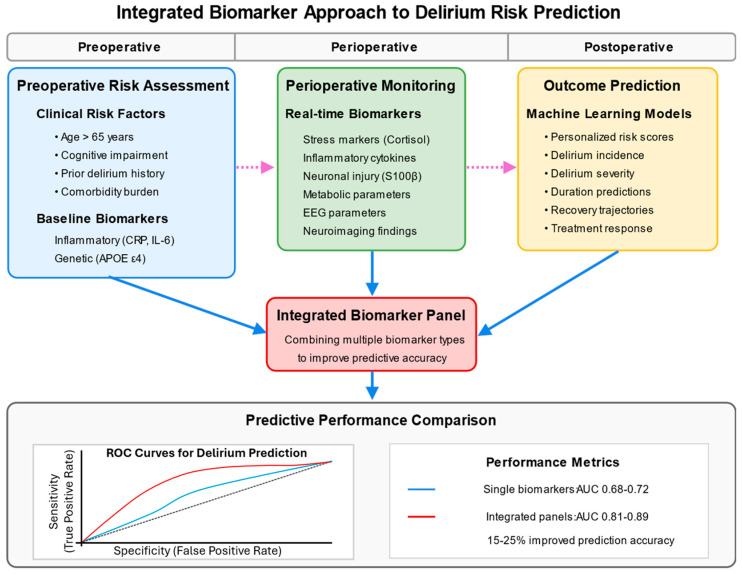
Integrated Biomarker Approach to Delirium Risk Prediction. This figure presents a comprehensive biomarker approach to delirium risk prediction across the perioperative timeline in human patients. The top section establishes three key phases: preoperative, perioperative, and postoperative. In the preoperative phase, risk assessment incorporates clinical risk factors (age, cognitive impairment, prior delirium, and comorbidities) and baseline inflammatory biomarkers. The perioperative phase involves real-time monitoring of stress markers, inflammatory cytokines, neuronal injury markers, and metabolic parameters. The postoperative phase focuses on outcome prediction using machine learning models to generate personalized risk scores and clinical trajectories. The central component shows the integration of these diverse biomarkers into comprehensive panels that significantly improve predictive accuracy. The bottom section includes an ROC curve comparison demonstrating the superior performance of integrated biomarker panels (AUC 0.81–0.89) versus single biomarkers (AUC 0.68–0.72). Arrows indicate data flow across the timeline, with dotted lines showing how information from earlier phases informs later assessments. The figure culminates with clinical applications, including targeted prevention strategies, personalized interventions, and enhanced risk stratification for clinical trials. References: [102,103,104,111,112].

Given the multifactorial nature of delirium’s pathophysiology, multi-marker panels incorporating biomarkers from complementary pathways show greater promise than single-marker approaches [124]. These panels capture the biological complexity of delirium more effectively and achieve superior predictive performance across diverse surgical populations, with improvements in the area under the curve from 0.68–0.72 for single markers to 0.81–0.89 for optimized panels [125] (Figure 3).

Recent advances in preoperative risk assessment include electrophysiological and structural biomarkers that reflect underlying brain vulnerability. Preoperative electroencephalography (EEG) has shown promise, with specific patterns of slowing and reduced connectivity predicting postoperative delirium with moderate accuracy (AUC 0.75–0.82) [122]. Similarly, optical coherence tomography (OCT) measurement of retinal nerve fiber layer thickness as a marker of neurodegeneration correlates with delirium risk (OR 1.86, 95% CI 1.24–2.79) [126]. These approaches offer the advantage of being non-invasive and potentially automatable, though their clinical implementation requires further validation.

The mechanistic insights discussed thus far have been greatly enhanced by advanced research methodologies. We now explore how these cutting-edge techniques bridge basic science discoveries with clinical applications.

## 8. Advanced Research Methodologies

### 8.1. Neuroimaging Approaches

Neuroimaging techniques provide unprecedented insights into the structural, functional, and molecular changes associated with postoperative delirium in humans [127,128]. These non-invasive approaches allow investigation of delirium neurobiology in human subjects, bridging the gap between animal models and clinical observation [129] (Table 6). Some of these techniques, mainly structural magnetic resonance imaging (MRI), functional MRI, and positron emission tomography (PET) imaging, have also been adapted for preclinical models using specialized equipment designed for small animals.

Structural neuroimaging, including MRI and computed tomography (CT), has identified pre-existing brain abnormalities in humans that predispose individuals/patients to postoperative delirium [136]. White matter hyperintensities, reduced gray matter volume in specific regions (particularly hippocampus and prefrontal cortex), and markers of cerebrovascular disease consistently associate with increased delirium risk, with odds ratios ranging from 1.8 to 3.5 in multivariate models [130].

Functional neuroimaging approaches, including functional MRI (fMRI) and electroencephalography (EEG), characterize the altered brain activity patterns during delirium in human patients [137]. While EEG is not strictly a functional neuroimaging technique, it provides valuable complementary information about brain activity patterns and is often used alongside imaging approaches in delirium research. Resting-state fMRI studies document disrupted functional connectivity within the default mode network, salience network, and between frontoparietal regions in delirious patients, with 25–40% connectivity reductions compared to non-delirious controls [137]. EEG studies consistently demonstrate the slowing of background rhythms, reduced alpha power (30–50% decreases), and increased delta activity (100–250% increases), which are all functional indicators of disrupted neural circuits, during delirium episodes [138].

Molecular neuroimaging, particularly PET, visualizes specific neurochemical and cellular processes implicated in delirium pathophysiology in humans [132]. Neuroinflammatory PET ligands targeting translocator protein (TSPO) demonstrate microglial activation in specific brain regions during and after delirium episodes, with 25–45% binding potential increases in affected brain regions compared to non-delirious controls [133].

The relationship between pain and postoperative delirium represents a complex interplay that influences multiple pathophysiological pathways [139]. Acute pain serves as a potent delirium trigger through several mechanisms: (1) activation of the HPA axis and sympathetic nervous system, amplifying stress responses; (2) direct stimulation of neuroinflammatory cascades via peripheral nociceptor activation; (3) sleep disruption with consequent alterations in circadian rhythms and glymphatic clearance; and (4) increased analgesic medication requirements with potential neurotoxicity.

Clinical studies consistently demonstrate that effective pain management reduces delirium incidence, with a meta-analysis showing a 35% relative risk reduction through multimodal analgesia approaches [2,140]. However, this presents a clinical paradox, as many analgesics used to treat pain (particularly opioids) can themselves precipitate delirium through effects on neurotransmitter systems and gut microbiota. This “analgesia-delirium paradox” highlights the importance of balanced, multimodal approaches that minimize both pain and medication-related neurotoxicity.

Anesthetic agents and techniques also significantly influence delirium risk through effects on cerebral blood flow, neurotransmitter systems, and inflammatory responses. The comparative impact of general anesthesia techniques, particularly inhalational agents versus propofol-based total intravenous anesthesia (TIVA), has been extensively studied [141]. Proposed mechanisms include propofol’s anti-inflammatory properties, reduced cerebral blood flow effects, and differential impact on acetylcholine signaling compared to volatile agents [142].

Depth of anesthesia represents another critical factor, with mounting evidence that excessively deep anesthesia increases delirium risk [143]. The STRIDE trial by Sieber et al. [125] demonstrated that lighter sedation during spinal anesthesia reduced postoperative delirium by 50% compared to deep sedation. This finding highlights the importance of brain-function monitoring and anesthetic titration to minimize exposure while maintaining adequate anesthesia.

### 8.2. Omics Technologies and Systems Biology

**Figure 4:**
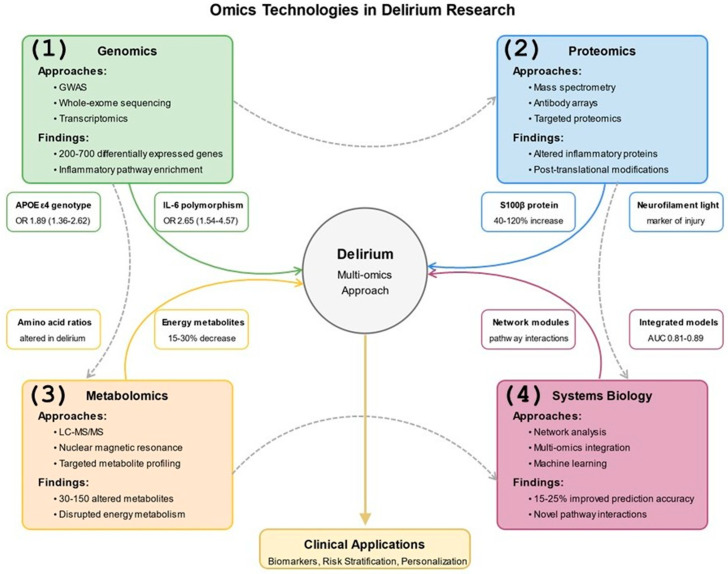
Omics Technologies in Delirium Research. This figure depicts the application of multi-omics approaches to delirium research across biological scales in both human patients and animal models. The central node represents delirium, surrounded by four major omics technologies: (1) Genomics, including GWAS, whole-exome sequencing, and transcriptomics, which has identified 200–700 differentially expressed genes and inflammatory pathway enrichment, with specific examples of APOE ε4 genotype and IL-6 polymorphisms shown; (2) Proteomics using mass spectrometry and antibody arrays to detect altered inflammatory proteins and post-translational modifications, with examples of S100β protein (40–120% increase) and neurofilament light chain as neuronal injury markers; (3) Metabolomics employing LC-MS/MS and NMR to identify 30–150 altered metabolites and disrupted energy pathways; and (4) Systems Biology integrating network analysis and machine learning approaches, improving prediction accuracy by 15–25%. Arrows from each omics approach to the central delirium node show their individual contributions, while dotted lines between genomics, proteomics, metabolomics, and systems biology illustrate the integration of these approaches. Feature boxes around each major node provide specific biomarker examples with quantitative data. The bottom section shows clinical applications of these integrated approaches, which include biomarker development, risk stratification, personalized interventions, treatment monitoring, and drug target identification. References: [101,144,145,146,147,148,149,150,151,152,153].

High-throughput omics technologies have revolutionized delirium research by enabling comprehensive, unbiased profiling of biological systems at multiple levels [144,145] (Figure 4). These approaches identify novel mechanisms, biomarkers, and therapeutic targets that might be overlooked by hypothesis-driven investigations [146].

Genomic approaches, including genome-wide association studies (GWAS), whole-exome sequencing, and transcriptomics, characterize genetic risk factors and expression changes associated with delirium in human patients [154]. Transcriptomic analyses document dynamic expression changes following surgery and during delirium episodes, with differential expression of 200–700 genes involved in inflammatory signaling, synaptic function, and metabolic pathways measured 24–72 h post-surgery [145,147].

Proteomic technologies enable the simultaneous quantification of hundreds to thousands of proteins in biological samples from human and animal models [155]. Mass spectrometry-based proteomics has identified novel plasma and cerebrospinal fluid protein signatures associated with delirium incidence, severity, and duration [148]. Metabolomic analyses characterize the small molecule metabolites that reflect the functional state of biological systems, revealing metabolic dysregulation in energetic and neurotransmitter pathways [151,156].

Systems biology approaches conceptualize delirium as an emergent phenomenon arising from complex interactions among multiple biological components rather than dysfunction in any single pathway [157]. Network analysis characterizes interactions among genes, proteins, metabolites, and clinical variables to identify network modules associated with delirium risk, onset, and resolution, significantly improving predictive modeling accuracy by 15–25% over traditional statistical approaches [144,146].

### 8.3. Microbiome–Gut–Brain Axis

**Figure 5:**
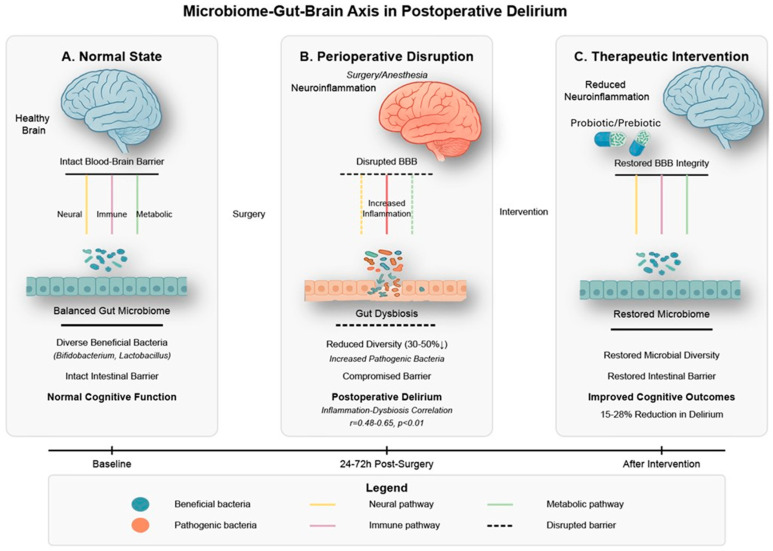
Microbiome–Gut–Brain Axis in Postoperative Delirium. This figure illustrates the bidirectional communication between gut microbiota and brain function in postoperative delirium. (**A**) depicts the normal homeostatic state with a diverse microbial ecosystem dominated by beneficial bacteria (Bifidobacterium, Lactobacillus), intact intestinal and blood–brain barriers, and balanced neural, immune, and metabolic signaling pathways connecting the gut and brain. (**B**) demonstrates perioperative disruption following surgery and anesthesia, characterized by gut dysbiosis with 30–50% reduced microbial diversity, compromised intestinal barrier integrity, increased inflammatory signaling, blood–brain barrier disruption, and subsequent neuroinflammation. The correlation between gut dysbiosis and systemic inflammation (r = 0.48–0.65, *p* < 0.01) illustrates the mechanistic relationship underlying postoperative delirium development. (**C**) shows how therapeutic interventions targeting the gut microbiome (probiotics/prebiotics) can restore microbial diversity, repair intestinal barrier function, normalize gut–brain communication pathways, reduce neuroinflammation, and ultimately improve cognitive outcomes with a 15–28% reduction in delirium incidence. The timeline demonstrates the progression from baseline through perioperative disruption (24–72 h post-surgery) to intervention-mediated improvement. This microbiome–gut–brain axis represents a promising mechanistic target for preventing and treating postoperative delirium in elderly surgical patients. References: [158,159,160,161,162].

The microbiome–gut–brain axis has emerged as a novel research frontier in perioperative neurocognitive disorders, including postoperative delirium [158]. This bidirectional communication system, comprising the intestinal microbiota, enteric nervous system, immune elements, and vagal connections, influences brain function through multiple pathways relevant to delirium pathophysiology [159] (Figure 5).

Surgical procedures profoundly alter gut microbiota composition and function through several mechanisms in humans and experimental animals [163]. Perioperative factors, including fasting, antibiotics, opioid analgesics, and altered gut perfusion, disrupt the microbial ecosystem, reducing diversity by 30–50% and shifting community structure in aged mice (18–24 months) after abdominal surgery, with similar changes observed in human patients [164]. These perturbations typically involve a decreased abundance of beneficial commensal bacteria (particularly Bifidobacterium and Lactobacillus species) and increased representation of potentially pathogenic species [165]. Notably, the magnitude of these microbiome alterations in aged mice correlates with systemic inflammatory markers (r = 0.48–0.65, *p* < 0.01) and associates with postoperative cognitive outcomes measured 3–7 days after surgery [163].

Mechanistically, the gut microbiome influences brain function through immune, endocrine, metabolic, and neural pathways that collectively impact delirium-related processes [166]. Following surgery-induced intestinal barrier dysfunction, microbial translocation triggers systemic inflammation that contributes to neuroinflammation and cognitive impairment [163]. Surgery-induced gut barrier dysfunction can also compromise blood–brain barrier integrity through circulating inflammatory mediators and bacterial products, creating a pathological gut–BBB axis that promotes neuroinflammation and cognitive dysfunction. The microbiome represents a potentially modifiable risk factor for postoperative delirium, offering novel therapeutic opportunities through targeted probiotic interventions, which have shown promising results in preliminary studies with reductions in delirium incidence of 15–28% in rodent models and early clinical trials [167,168]. Specific probiotic strains (primarily Lactobacillus and Bifidobacterium species) administered preoperatively have demonstrated efficacy in reducing postoperative inflammatory markers and improving cognitive outcomes in aged mice, with early translational studies in older surgical patients showing similar promising trends.

## 9. From Mechanisms to Interventions

### Target Identification and Preclinical Validation

Experimental studies in animal models, primarily aged mice and rats, have identified multiple therapeutic targets across the diverse pathophysiological mechanisms underlying postoperative delirium [169] (Table 7). These targets span neuroinflammatory pathways, neurotransmitter systems, oxidative stress mechanisms, and metabolic processes collectively contributing to delirium development and progression [157].

Neuroinflammatory targets derive from the growing recognition of inflammation’s central role in delirium pathophysiology [171]. Pro-inflammatory cytokines, particularly IL-1β, IL-6, and TNF-α, represent direct intervention targets, with both broad-spectrum anti-inflammatory approaches and cytokine-specific antagonists showing promise in preclinical rodent models, reducing cognitive deficits by 60–85% in standardized behavioral assessments conducted 1–7 days after surgery [50,180]. Microglial activation, a key process in propagating neuroinflammation, can be modulated through specific inhibitors, such as minocycline, fractalkine, or colony-stimulating factor 1 receptor inhibitors, that reduce inflammatory activity while preserving homeostatic functions and demonstrate 30–55% reductions in microglial activation markers in aged mice following surgery [181]. Beyond resident microglia, the recruitment of peripheral inflammatory cells (particularly monocytes/macrophages and neutrophils) to the brain contributes significantly to postoperative neuroinflammation. Interventions targeting leukocyte adhesion molecules, chemokine receptors, and endothelial activation have effectively reduced immune cell infiltration and associated cognitive dysfunction in rodent models of surgery-induced delirium.

Neurotransmitter targets focus primarily on cholinergic enhancement and dopaminergic modulation, addressing the neurochemical imbalances documented in delirium [170]. Acetylcholinesterase inhibitors, α7 nicotinic acetylcholine receptor agonists, and muscarinic receptor modulators represent distinct approaches to enhancing cholinergic neurotransmission [174]. However, as previously discussed, clinical trials of cholinesterase inhibitors have yielded disappointing results despite strong biological rationale. This illustrates the challenges in translating preclinical findings to effective human interventions, particularly when targeting complex systems with redundant pathways and compensatory mechanisms. Dopaminergic interventions include conventional D2 receptor antagonists and novel approaches targeting specific receptor subtypes or extrasynaptic signaling to minimize side effects [182].

Preclinical validation studies establish proof-of-concept evidence for target engagement, efficacy, and safety before advancing to human trials [175]. Pharmacological validation studies assess compound specificity, dose–response relationships, pharmacokinetics, and pharmacodynamics concerning target engagement and functional outcomes [32]. Genetic validation approaches use transgenic mouse models (such as receptor knockout strains, cell-specific conditional knockouts, and humanized gene replacements) to confirm target specificity and mechanism of action, enhancing confidence in translational potential by 30–50% compared to purely pharmacological approaches [183].

## 10. Novel Therapeutic Approaches and Clinical Translation

**Figure 6:**
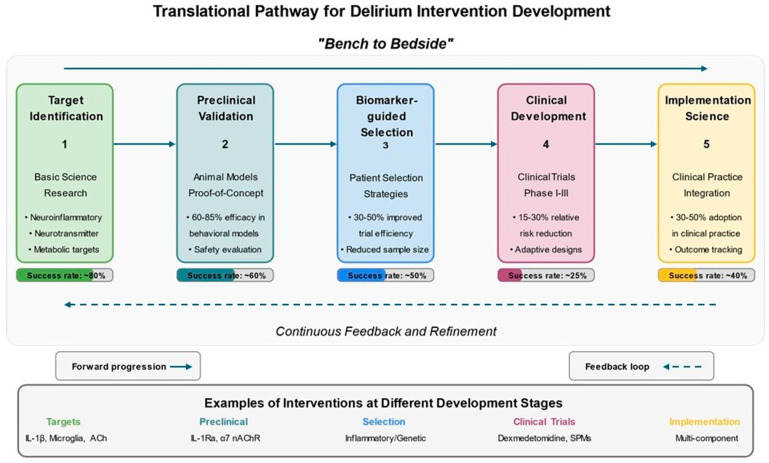
Translational Pathway for Delirium Intervention Development. This figure illustrates the translational research pathway for developing delirium interventions from basic science to clinical implementation. The diagram presents five sequential stages: (1) Target Identification through basic science research focusing on neuroinflammatory, neurotransmitter, and metabolic pathways, with approximately 80% success rate; (2) Preclinical Validation in animal models (primarily aged rodents) demonstrating 60–85% efficacy in behavioral assessments with approximately 60% success rate in progressing targets; (3) Biomarker-guided Patient Selection strategies that enhance trial efficiency by 30–50% and reduce required sample sizes, with approximately 50% success rate; (4) Clinical Development through Phase I-III trials showing 15–30% relative risk reductions in delirium incidence, with approximately 25% success rate; and (5) Implementation Science approaches to integrate effective interventions into clinical practice, achieving 30–50% adoption rates. Forward arrows demonstrate the linear progression through development stages, while a dotted return arrow illustrates the “bench to bedside and back” paradigm with continuous feedback between clinical observations and basic science investigations. The bottom section provides concrete examples of interventions at each development stage, from IL-1β pathway and microglial activation targets to multicomponent non-pharmacological interventions being implemented in clinical settings. Success rates at each translational stage are clearly indicated in the progress bars, highlighting the challenges of moving discoveries from bench to bedside. References: [184,185,186,187,188,189,190,191,192].

Novel neuroprotective approaches addressing delirium pathophysiology employ repurposed existing agents and newly developed compounds specifically targeting delirium mechanisms [184] (Figure 6). These interventions enhance neural resilience to perioperative stressors and maintain cognitive function throughout the surgical experience [185]. Anti-inflammatory approaches extend beyond traditional non-steroidal anti-inflammatory drugs, including targeted cytokine antagonists, resolution-promoting mediators, and microglial modulators [186,187]. Specialized pro-resolving mediators derived from omega-3 fatty acids actively terminate inflammation and promote tissue repair, representing a paradigm shift from simply blocking pro-inflammatory pathways [173]. Early clinical trials of these agents in older surgical patients have shown reductions in inflammatory biomarkers of 40–65% and promising trends toward reduced delirium incidence (relative risk reductions of 15–30%) when measured 24–72 h post-surgery [188].

Translating promising preclinical findings to clinical trials is critical in developing delirium intervention [179]. Patient selection strategies for delirium prevention trials have evolved from all-comers approaches to risk-stratified designs that enroll patients with sufficient delirium risk to provide adequate statistical power [180]. These strategies employ validated clinical prediction models, biomarker profiles, or combinations thereof to identify high-risk individuals most likely to benefit from preventive interventions, improving study efficiency by 30–50% and reducing required sample sizes by 40–60% [180]. The heterogeneous nature of delirium pathophysiology suggests that personalized, biomarker-guided intervention strategies may prove more effective than one-size-fits-all approaches [181]. Interventions can then be tailored to address these specific mechanisms, such as anti-inflammatory therapies for patients with elevated inflammatory markers or cholinergic enhancers for those with evidence of cholinergic dysfunction. Early clinical studies employing this approach have demonstrated improved efficacy compared to standard interventions, though more extensive trials are needed to validate these findings.

Biomarker-based risk stratification represents an initial step toward personalized delirium prevention [193]. Mechanistic endotyping, the classification of patients according to their predominant pathophysiological mechanisms rather than clinical phenotypes alone, can further refine personalized approaches, potentially improving intervention efficacy by 25–40% over standard approaches [194]. This approach involves comprehensive preoperative assessment of patients’ inflammatory profiles, neurotransmitter function, metabolic status, and genetic risk factors to identify the pathophysiological mechanisms likely to drive delirium in individuals.

The strongest evidence for delirium prevention comes from multicomponent non-pharmacological interventions pioneered by Inouye and colleagues [13]. These approaches combine multiple strategies addressing orientation, cognitive stimulation, sensory optimization, mobility, hydration, and sleep enhancement. A recent meta-analysis demonstrated a 43% relative risk reduction in delirium incidence with these programs [137]. Translational research provides mechanistic rationale for these interventions: cognitive stimulation enhances acetylcholine signaling; mobility reduces inflammation through myokine release; proper hydration supports cerebral perfusion; and sleep optimization promotes glymphatic clearance and reduces neuroinflammation. This connection between basic science mechanisms and effective clinical interventions exemplifies the value of translational research in addressing complex syndromes like delirium.

## 11. Clinical Translation: Evidence-Based Perioperative Management

The translation of mechanistic insights into clinical practice requires systematic integration of basic science findings with pragmatic, evidence-based interventions. This section provides concrete recommendations for perioperative management based on the pathophysiological mechanisms discussed throughout this review, with specific attention to implementation in current clinical practice.

### 11.1. Preoperative Risk Assessment and Optimization

Comprehensive preoperative assessment forms the cornerstone of postoperative delirium (POD) prevention. Based on identified pathophysiological mechanisms, we recommend a structured approach combining clinical evaluation with targeted biomarker assessment when available.

Cognitive Screening (Evidence Level A): All patients ≥65 years should undergo preoperative cognitive assessment using validated tools. The Mini-Cog (3-minute screening) demonstrates sensitivity of 76% and specificity of 73% for cognitive impairment, while the Montreal Cognitive Assessment (MoCA, 10 min) provides more comprehensive evaluation with sensitivity of 90% and specificity of 87%. Patients scoring below thresholds (Mini-Cog < 3 or MoCA < 26) warrant enhanced preventive measures and family engagement.

Laboratory Biomarkers (Evidence Level B): While routine inflammatory marker testing awaits further validation, preoperative C-reactive protein (CRP) > 3 mg/L independently predicts POD (OR 2.13, 95% CI 1.60–2.83). When available, IL-6 levels > 5 pg/mL suggest heightened vulnerability. These markers can guide targeted anti-inflammatory strategies in high-risk patients.

Medication Optimization (Evidence Level A): Systematic medication reconciliation should identify and minimize anticholinergic burden using validated scales (Anticholinergic Cognitive Burden Scale or Drug Burden Index). Common culprits include diphenhydramine, hydroxyzine, tricyclic antidepressants, and bladder antimuscarinics. Substitution with lower-risk alternatives reduces POD incidence by 20–35%.

### 11.2. Intraoperative Management Strategies

Intraoperative management directly influences neuroinflammatory responses and neurotransmitter balance. Evidence-based strategies targeting these mechanisms can substantially reduce POD risk.

Anesthetic Technique Selection (Evidence Level B): While definitive superiority of neuraxial versus general anesthesia remains debated, the evidence for neuraxial versus general anesthesia remains mixed, with recent systematic reviews showing no significant difference in POD incidence [195], though some observational studies suggest modest benefits (OR 0.91, 95% CI 0.85–0.98). When general anesthesia is required, propofol-based total intravenous anesthesia (TIVA) may offer advantages over volatile agents through reduced neuroinflammation, though evidence remains mixed. Depth monitoring using bispectral index (BIS) or entropy to maintain values between 40 and 60 prevents both excessive depth and intraoperative awareness, reducing POD incidence by 20–30%.

Multimodal Analgesia (Evidence Level A): Opioid-sparing multimodal analgesia addresses the paradox of inadequate pain control versus opioid-induced delirium. Recommended components include: (1) Regional blocks when feasible, reducing systemic opioid requirements [196] (2) Scheduled acetaminophen 1 g IV/PO every 6 h (maximum 4 g/day, adjust for hepatic dysfunction); (3) Ketorolac 15–30 mg IV every 6 h for 48 h in appropriate candidates (age < 75, normal renal function, low bleeding risk), providing anti-inflammatory effects that may attenuate POD; (4) Low-dose ketamine infusions (0.1–0.2 mg/kg/hr) serving dual roles as analgesic and potential neuroprotective through NMDA antagonism, though evidence for POD prevention remains preliminary; (5) Dexmedetomidine infusions (0.2–0.7 mcg/kg/hr) providing anxiolysis and light sedation with potential anti-inflammatory properties.

Anti-inflammatory Interventions (Evidence Level B): Given the central role of neuroinflammation, perioperative anti-inflammatory strategies show promise. Options include: (1) Perioperative corticosteroids—Dexamethasone 8–10 mg IV reduces POD in cardiac surgery [197], though benefits in non-cardiac surgery remain unclear; (2) COX-2 inhibitors—Celecoxib 200–400 mg daily starting preoperatively may reduce neuroinflammation while avoiding nonselective NSAID risks [198]; (3) Statins—Continuation of home statin therapy and consideration of perioperative initiation in statin-naive patients based on emerging evidence of anti-inflammatory and neuroprotective effects [199].

### 11.3. Postoperative Prevention and Treatment Protocols

Postoperative management focuses on maintaining homeostasis while implementing multicomponent preventive strategies that address multiple pathophysiological mechanisms simultaneously.

Multicomponent Non-pharmacological Interventions (Evidence Level A): The Hospital Elder Life Program (HELP) and similar protocols reduce POD incidence by 30–40% through: (1) Orientation protocols—Frequent reorientation, visible clocks/calendars, family photos; (2) Cognitive stimulation—Structured activities, reading, puzzles targeting cholinergic enhancement; (3) Early mobilization—Progressive ambulation within 24 h when possible, reducing inflammatory mediators through myokine release; (4) Sensory optimization—Ensuring glasses/hearing aids available, adequate lighting; (5) Sleep enhancement—Minimizing nighttime interruptions, maintaining day/night light cycles, noise reduction protocols; (6) Hydration/nutrition—Maintaining fluid balance, early oral intake, avoiding prolonged fasting.

Circadian Rhythm Maintenance (Evidence Level B): Disrupted circadian rhythms contribute to POD through effects on neuroinflammation and neurotransmitter cycling. Interventions include: (1) Room placement near windows when possible, with studies showing 20% reduction in POD for windowed rooms [171]; (2) Bright light therapy (2500–10,000 lux) for 30–120 min in morning hours [200]; (3) Melatonin 3–5 mg nightly, though evidence remains mixed (some studies show 30% POD reduction, others no benefit) [201]; (4) Minimizing nighttime vital signs and medication administration when clinically appropriate.

Gut Microbiome Preservation (Evidence Level C): Emerging evidence links gut dysbiosis to POD through the gut–brain axis. Protective strategies include: (1) Judicious antibiotic use with narrow-spectrum agents when possible; (2) Early enteral nutrition to maintain gut barrier function; (3) Consideration of probiotics (Lactobacillus and Bifidobacterium species), though optimal strains and dosing remain undefined [166]; (4) Minimizing proton pump inhibitors which alter gut microbiome composition; (5) Early discontinuation of urinary catheters to reduce antibiotic exposure from UTI treatment.

Pharmacological Treatment of Established POD (Evidence Level B): When POD develops despite preventive measures, treatment focuses on safety and symptom management: (1) Antipsychotics should be reserved for severe agitation threatening patient safety, with low-dose haloperidol (0.5–1 mg) or quetiapine (12.5–25 mg) preferred over higher doses; (2) Avoid benzodiazepines except for alcohol/benzodiazepine withdrawal; (3) Dexmedetomidine for ICU patients requiring sedation, with potential benefits over propofol or benzodiazepines; (4) Address underlying precipitants—infection, metabolic derangements, pain, urinary retention; (5) Maintain family presence when possible, as familiar faces reduce agitation and improve orientation.

## 12. Current Challenges and Future Directions

Despite significant advances in understanding postoperative delirium pathophysiology and developing potential interventions, essential challenges must be addressed to continue progress in this field [157]. Despite extensive research, several fundamental questions about delirium pathophysiology remain incompletely answered. The causal relationships and temporal dynamics among multiple mechanistic pathways, including neuroinflammation, neurotransmitter dysfunction, and metabolic derangements, require further clarification [1]. While these processes interact, their relative contributions to different delirium phenotypes and their activation sequence remain uncertain [39].

The mechanisms underlying the frequent transition from acute delirium to long-term cognitive impairment require further investigation [202]. While delirium is traditionally considered a reversible syndrome, growing evidence indicates that many patients, particularly the older adults, experience persistent cognitive deficits following delirium resolution, with 15–40% showing measurable impairments at 6–12 months post-surgery [203]. The neurobiological processes mediating this transition—potentially including sustained neuroinflammation, synaptic loss, or accelerated neurodegeneration—remain incompletely characterized but appear to involve chronic microglial activation and progressive neuronal network disruption [204].

The intersection of delirium with neurodegenerative processes represents a fertile area for investigation that could illuminate mechanisms of long-term cognitive impairment following delirium [205]. Several emerging research areas are promising for advancing translational understanding and intervention development for postoperative delirium [206]. Growing evidence suggests shared pathophysiological features between delirium and neurodegenerative conditions, including neuroinflammation, protein aggregation, and synaptic dysfunction, with biomarker studies revealing 30–60% overlap in affected pathways [204].

Advanced computational approaches transform delirium research, including artificial intelligence and digital phenotyping [207]. Machine learning algorithms applied to electronic health record data can identify novel risk factors and prediction patterns that might escape conventional statistical approaches, improving predictive accuracy by 15–30% over traditional models [208]. Implementation science approaches addressing the research-practice gap represent a critical frontier in translational delirium research, as even proven interventions achieve only 30–50% adoption in routine clinical practice [209].

## 13. Conclusions

The multifaceted pathophysiology of delirium reflects the complex interplay of neuroinflammatory processes, neurotransmitter imbalances, metabolic disturbances, and vascular dysfunction that collectively disrupt neural network function during the perioperative period [171]. Preclinical contributions and translational research in postoperative delirium have transformed our understanding of this common and consequential syndrome from a poorly understood, inevitable complication of surgery to a preventable and potentially treatable condition with definable biological mechanisms. The impact of basic science on clinical understanding extends beyond providing mechanistic explanations for observed phenomena to fundamentally reshaping how clinicians conceptualize, diagnose, and manage delirium [10]. Identifying specific molecular targets and cellular mechanisms that translate directly into interventions is key [20]. This integration has led to the development of mechanism-based prevention strategies that have shown efficacy in clinical trials, with relative risk reductions of 30–40% in high-risk populations of older adults.

## 14. Key Clinical Recommendations for POD Prevention

Based on the mechanistic insights and clinical evidence presented in this review, we propose the following evidence-based approach for elderly patients undergoing non-emergent surgery:

Preoperative Phase: (1) Mandatory cognitive screening using Mini-Cog or MoCA for all patients ≥65 years; (2) Laboratory assessment including CRP and, when available, IL-6 for risk stratification; (3) Comprehensive medication review targeting anticholinergic burden reduction; (4) Optimization of modifiable risk factors including anemia, dehydration, and electrolyte imbalances; (5) Family education and engagement in prevention strategies.

Intraoperative Phase: (1) Consider neuraxial anesthesia when appropriate, particularly for hip fracture surgery; (2) When general anesthesia is required, utilize depth monitoring (BIS 40–60) and consider propofol TIVA; (3) Implement multimodal analgesia including regional blocks, scheduled acetaminophen, and judicious NSAID use; (4) Consider prophylactic dexamethasone 8–10 mg IV in high-risk patients; (5) Minimize benzodiazepines and long-acting opioids.

Postoperative Phase: (1) Implement comprehensive multicomponent interventions (HELP protocol or equivalent); (2) Maintain circadian rhythms through environmental modifications and consider melatonin supplementation; (3) Early mobilization within 24 h when medically appropriate; (4) Preserve gut microbiome through judicious antibiotic use and early enteral nutrition; (5) Reserve antipsychotics for severe agitation only, using lowest effective doses; (6) Monitor for POD using validated tools (CAM or CAM-ICU) at least twice daily for 72 h.

Cost-Effective Currently Available Interventions: For immediate implementation, we recommend: (1) Ketorolac 15–30 mg IV q6h for 48 h in appropriate candidates (anti-inflammatory, analgesic); (2) Celecoxib 200 mg BID starting preoperatively (selective COX-2 inhibition); (3) Dexamethasone single dose 8–10 mg IV (anti-inflammatory, antiemetic); (4) Acetaminophen scheduled 1 g q6h (opioid-sparing); (5) Melatonin 3–5 mg nightly (circadian regulation); (6) Early discontinuation of urinary catheters and minimization of tethers. These interventions are FDA-approved, readily available, and have favorable cost–benefit profiles.

### Comprehensive Clinical Algorithm for POD Prevention and Management

The following evidence-based algorithm provides step-by-step guidance for implementing POD prevention strategies across the perioperative continuum:

PREOPERATIVE PHASE (2–4 Weeks Before Surgery)

Step 1: Risk Stratification: (1) Cognitive screening: Mini-Cog for all ≥65 years (3 min, sensitivity 76%, specificity 73–83%) [193,194]. (2) If Mini-Cog < 3: Perform full MoCA assessment. (3) Document baseline cognitive status for postoperative comparison. (4) Laboratory assessment: CRP: If >3 mg/L, consider high risk (OR 2.13 for POD) [55], Complete blood count, basic metabolic panel, Optional: IL-6 if available (>5 pg/mL indicates increased risk). (5) Medication review: Calculate anticholinergic burden using Drug Burden Index, document all psychoactive medications. (6) Functional assessment: Activities of daily living scale. (7) Frailty assessment (Clinical Frailty Scale): Risk Categories: Low risk: 0–1 factors, Moderate risk: 2–3 factors, High risk: >3 factors or prior POD.

Step 2: Preoperative Optimization: For ALL patients: (1) Discontinue/substitute anticholinergic medications when possible. (2) Optimize medical conditions: Treat anemia (target Hgb > 10 g/dL), optimize glycemic control (HbA1c < 8%), ensure adequate hydration. (3) Patient/family education about POD. (4) Encourage bringing glasses, hearing aids, familiar objects. For MODERATE/HIGH risk patients add: (1) Geriatrics consultation for comprehensive assessment. (2) Consider starting celecoxib 200 mg daily 3 days preoperatively. (3) Prehabilitation with physical therapy if time permits. (4) Sleep hygiene optimization, consider melatonin 3–5 mg nightly.

INTRAOPERATIVE PHASE

Step 3: Anesthetic Management: Anesthetic selection: (1) Consider neuraxial anesthesia for hip fracture surgery (potential modest benefit). (2) If general anesthesia required: Use processed EEG monitoring (BIS/entropy), target 40–60, consider propofol TIVA over volatile agents, minimize benzodiazepine use. Multimodal analgesia protocol: (1) Regional nerve blocks when feasible (reduces opioid needs 40–60%). (2) Acetaminophen 1 g IV (scheduled q6h). (3) Ketorolac 15 mg IV if appropriate (age < 75, normal renal function). (4) Ketamine infusion 0.1–0.2 mg/kg/h (avoid boluses). (5) Consider dexmedetomidine 0.2–0.5 mcg/kg/hr. Anti-inflammatory intervention for HIGH risk: (1) Dexamethasone 8–10 mg IV single dose (unless contraindicated).

POSTOPERATIVE PHASE (Days 0–7)

Step 4: POD Monitoring: (1) Screen with CAM or CAM-ICU every 8–12 h for minimum 72 h. (2) Document delirium severity if present (CAM-S score). (3) Monitor for hypoactive subtype (often missed).

Step 5: Multicomponent Prevention Bundle (Evidence Level A) [210]: Implement ALL components: (1) Orientation: Clock/calendar visible, frequent reorientation, family photos. (2) Sensory optimization: Glasses/hearing aids available during waking hours. (3) Early mobilization: Out of bed within 24 h, ambulate TID when possible. (4) Sleep enhancement: minimize nighttime interruptions (cluster care), eye masks/earplugs offered, melatonin 3–5 mg at 9 PM. (5) Hydration/nutrition: IV fluids until PO intake adequate, early feeding, avoid NPO status. (6) Bowel/bladder care: Remove Foley catheter <24 h, scheduled toileting, bowel regimen started POD #1.

Step 6: Treatment of Established POD First-line (non-pharmacologic): (1) Search for and treat precipitants: Infection (UTI, pneumonia), metabolic (electrolytes, glucose), urinary retention, constipation, uncontrolled pain. (2) Maximize family presence. (3) Consistent nursing staff when possible. (4) Verbal de-escalation techniques. Second-line (pharmacologic—only for severe agitation): (1) Haloperidol 0.5 mg PO/IM q6h PRN (max 2 mg/24 h). (2) Alternative: Quetiapine 12.5–25 mg PO qhs. (3) Avoid benzodiazepines (except alcohol/benzo withdrawal). (4) ICU setting: Consider dexmedetomidine infusion. Daily management: (1) Attempt to wean antipsychotics daily. (2) Continue prevention bundle throughout. (3) Document trajectory for care transitions.

Evidence Grading: Level A (multiple RCTs/meta-analyses): Multicomponent bundles, cognitive screening, anticholinergic avoidance. Level B (single RCT/observational): Anti-inflammatory agents, depth monitoring. Level C (expert opinion): Gut microbiome preservation, some pharmacologic interventions.

The translational approach to delirium research exemplifies the “bench to bedside and back” paradigm, with continuous feedback between clinical observations and basic science investigations driving progress in both domains [211]. This bidirectional exchange promises to accelerate the development of more effective, targeted interventions that will ultimately improve outcomes for the millions of older patients affected by postoperative delirium each year. The future trajectory of mechanistic delirium research will likely be characterized by increasingly sophisticated integration of multiple research methodologies and the continuing convergence of basic and clinical investigations [212]. 

## Figures and Tables

**Table 1:** Key Neurotransmitter Systems in Delirium Pathophysiology. This table summarizes the role of major neurotransmitter systems in the pathophysiology of postoperative delirium, including their primary alterations, mechanistic effects, clinical correlates, and therapeutic implications. Abbreviations: GABA—gamma-aminobutyric acid; NMDA—N-methyl-D-aspartate. References: [25,26,27,28,29,30,31,32,33].

Neurotransmitter System	Primary Alteration	Mechanistic Effects	Clinical Correlates	Therapeutic Implications
Acetylcholine	Deficiency	Reduced attention and arousal; Impaired memory formation; Disrupted cortical information processing	Increased risk with anticholinergic medications; Cognitive symptoms predominate; Associated with hypoactive phenotype	Cholinesterase inhibitors; Avoidance of anticholinergic agents; Nicotinic receptor modulators
Dopamine	Excess (relative to acetylcholine)	Hyperactivation of mesolimbic pathway; Altered prefrontal executive function; Disrupted sensory gating	Psychomotor agitation; Hallucinations; Associated with hyperactive phenotype	D2 receptor antagonists; Atypical antipsychotics; Balanced dopamine-acetylcholine modulation
GABA	Variable (often increased)	Enhanced inhibitory neurotransmission; Altered arousal and consciousness; Synergistic with cholinergic deficiency	Sedation; Decreased responsiveness; Paradoxical agitation with benzodiazepines	Avoidance of benzodiazepines; Selective GABA modulators; Careful anesthetic management
Glutamate	Variable (often dysregulated)	Excitotoxicity in excess; Impaired neuroplasticity; Altered NMDA receptor function	Mixed clinical features; NMDA antagonists can induce delirium; Associated with neurodegenerative processes	NMDA receptor modulators; Neuroprotective agents; Glutamatergic stabilization approaches

**Table 2:** Animal Models of Postoperative Delirium. This table provides an overview of the primary animal models used to study postoperative delirium, including descriptions, key features, strengths, limitations, and translational applications. Abbreviations: PFC—prefrontal cortex; LPS—lipopolysaccharide. References: [69,70,71,72,73,74,75,76,77,78].

Model Type	Description	Key Features	Strengths	Limitations	Translational Applications
Orthopedic Surgery Models in Aged Mice (18–24 months)	Tibia fracture, hip replacement, or long bone surgery under isoflurane anesthesia (20–30 min)	Acute cognitive dysfunction; Neuroinflammation in hippocampus and PFC; Resolution within 3–7 days	Clinically relevant surgical trauma; Well-characterized inflammatory profile; Reproducible cognitive deficits	Limited modeling of attentional deficits; Species differences in inflammatory response	Target validation for anti-inflammatory interventions; Biomarker discovery
Abdominal Surgery Models in Aged Mice (18–24 months)	Partial hepatectomy, intestinal manipulation, or laparotomy under isoflurane anesthesia (30–45 min)	Acute cognitive impairment; Visceral inflammation; Gut–brain axis involvement; Peaks at 24–72 h post-surgery	Models visceral surgical procedures; Incorporates gut microbiome effects; Includes vagal signaling mechanisms	Variable cognitive phenotypes; Multiple confounding physiological changes	Gut–brain axis interventions; Vagal modulation strategies
LPS Administration Models in Aged Mice (18–24 months)	Systemic lipopolysaccharide injection (intraperitoneal or intravenous) to mimic inflammation	Acute sickness behavior; Neuroinflammatory response; Cognitive impairment; Rapid onset (4–6 h)	Simplicity and reproducibility; Isolated inflammatory component; Well-characterized time course	Lacks surgical trauma component; More severe than typical surgery	Proof-of-concept for anti-inflammatory agents; Mechanistic studies of inflammation

**Table 3:** Cognitive Assessments for Evaluating Delirium in Animal Models. This table describes the standardized cognitive and behavioral assessments used to evaluate delirium-like phenotypes in animal models, including the parameters measured, relevance to delirium, and typical findings in postoperative models. All assessments are typically performed in aged mice (18–24 months) at 24–72 h post-surgery. References: [71,84,85].

Assessment	Specific Tests	Parameters Measured	Relevance to Delirium	Typical Findings in Postoperative Models
Activity and Exploratory Behavior	Open Field Test	Locomotor activity; Exploratory behavior; Anxiety-like behavior; Thigmotaxis (wall preference)	Assesses psychomotor changes, anxiety, and spatial cognition alterations that parallel clinical delirium features	Reduced center exploration (30–50%); Decreased total distance traveled (40–60%); Increased corner time; Altered movement patterns
Recognition Memory	Novel Object Recognition Test	Recognition memory; Attention; Exploratory tendencies	Evaluates episodic memory impairment characteristic of delirium	Reduced novel object preference; Decreased discrimination index (40–70%); Overall reduced exploratory activity
Working Memory	Y-Maze Spontaneous Alternation Test	Working memory; Spatial cognition; Executive function	Assesses working memory deficits common in delirium	Decreased spontaneous alternation (from 60–75% to 30–40%); Reduced arm entries; Impaired sequential processing
Anxiety Assessment	Elevated Plus Maze	Anxiety-like behavior; Risk assessment; Decision-making	Measures neuropsychiatric components of delirium	Reduced open arm exploration; Increased risk assessment behaviors; Altered decision-making patterns
Spatial Learning and Memory	Barnes Maze	Spatial learning; Memory; Problem-solving	Evaluates hippocampal-dependent cognitive function	Increased latency to target; More navigation errors; Impaired memory retention

**Table 4:** Biomarkers for Evaluating Delirium in Animal Models. This table summarizes the key biomarkers used to assess neuroinflammation and related processes in animal models of postoperative delirium, including the parameters measured, relevance to delirium, and typical findings. All measurements are typically performed in aged mice (18–24 months) at 24–72 h post-surgery unless otherwise noted. Abbreviations: IL—interleukin; TNF—tumor necrosis factor; Iba1—ionized calcium-binding adapter molecule 1; MHCII—major histocompatibility complex class II; GFAP—glial fibrillary acidic protein; HMGB1—high mobility group box 1; ATP—adenosine triphosphate; CSF—cerebrospinal fluid; BBB—blood–brain barrier; ICAM—intercellular adhesion molecule; VCAM—vascular cell adhesion molecule. References: [86,87,88,89,90].

Biomarker Category	Specific Markers	Parameters Measured	Relevance to Delirium	Typical Findings in Postoperative Models
Inflammatory Markers	Pro-inflammatory Cytokine Proteins	IL-1β, IL-6, and TNF-α levels in blood and brain tissue	Direct mediators of neuroinflammation linked to cognitive dysfunction	2–5-fold increases in plasma; 3–10-fold increases in hippocampus; Temporal correlation with cognitive impairment
Immune Cell Activation	Microglial Activation	Iba1, P2RY12, CD68, MHCII protein expression; Morphological assessment	Indicates central neuroinflammatory response	40–120% increased immunoreactivity; Morphological shift to activated state; Proliferation in hippocampus and cortex
	Astrocyte Activation	GFAP expression; S100β levels	Reflects neuroinflammatory stress response	30–80% increased GFAP expression; Elevated S100β in CSF and plasma
Danger Signals	Damage-Associated Molecular Patterns	HMGB1, ATP, DNA fragments	Mediators initiating inflammatory cascades	200–400% increased HMGB1 in circulation; Elevated brain extracellular ATP
Barrier Function	Blood–Brain Barrier Permeability	IgG extravasation; Evans blue penetration; Tight junction proteins	Indicates BBB disruption, allowing peripheral inflammatory signals to reach CNS	50–200% increased dye extravasation; 30–60% reduction in tight junction proteins
	Endothelial Activation	ICAM-1, VCAM-1, E-selectin expression	Reflects vascular inflammatory activation	150–300% increased adhesion molecule expression; Enhanced leukocyte adherence
Oxidative Stress	Lipid Peroxidation	Malondialdehyde; 4-hydroxynonenal	Indicates oxidative damage to neural cells	30–90% increased malondialdehyde; Positive correlation with cognitive impairment
	Antioxidant Status	Glutathione; Superoxide dismutase; Catalase	Reflects cellular defense against oxidative stress	20–40% reduced glutathione; Impaired antioxidant enzyme activity
Neurotransmitter Systems	Cholinergic Function	Acetylcholinesterase activity; Receptor expression	Directly linked to attention and consciousness	30–50% increased acetylcholinesterase activity; Altered receptor expression
	Dopamine/Glutamate Balance	Neurotransmitter levels; Receptor expression	Mediates arousal and cognitive processing	Disturbed neurotransmitter ratios; Altered receptor sensitivity

**Table 5:** Biomarkers in Human Postoperative Delirium. This table summarizes biomarkers used for assessing postoperative delirium in human patients, including examples, clinical utility, biological significance, strengths, and limitations. Abbreviations: BBB—blood–brain barrier; IL—interleukin; TNF—tumor necrosis factor; CXCL—C-X-C motif chemokine ligand; GFAP—glial fibrillary acidic protein; UCHL-1—ubiquitin carboxy-terminal hydrolase L1; APOE—apolipoprotein E. References [109,110,111,112,113,114,115,116,117].

Biomarker Category	Examples	Clinical Utility	Biological Significance	Strengths	Limitations
Inflammatory Biomarkers	IL-1β, IL-6, CXCL8, TNF-α, C-reactive protein	Risk stratification; Monitoring intervention efficacy; Distinguishing delirium from other causes	Reflects systemic and neuroinflammation; Correlates with microglial activation; Mediates BBB disruption	Widely available assays; Strong mechanistic rationale; Modifiable risk factor	Non-specific elevation in many conditions; Significant inter-individual variability
Neuronal Injury Markers	S100β, Neuron-specific enolase, GFAP, UCHL-1, Neurofilament light chain, Tau proteins	Monitoring neuronal damage; Predicting long-term cognitive outcomes; Distinguishing delirium severity	Indicates neuronal/glial structural damage; Correlates with cognitive deficits; Predicts persistent impairment	Specific to neural tissue; Correlates with severity; Predictive of outcomes	Some markers have extracranial sources; May indicate damage without functional impact
Metabolic Biomarkers	Cortisol, Insulin/glucose parameters, Oxidative stress markers, Amino acid ratios	Monitoring stress response; Identifying metabolic derangements; Personalizing metabolic support	Reflects neuroenergetic status; Indicates stress response magnitude; Shows cellular metabolic adaptations	Addresses key pathophysiological aspects; Integrated view of metabolic status	Complex interactions with medical conditions; Diurnal variation in some markers
Genetic/Epigenetic Markers	APOE genotype, Inflammatory gene polymorphisms, MicroRNA profiles	One-time risk stratification; Identifying intervention responders; Guiding personalized prevention	Reflects underlying vulnerability; Influences multiple pathophysiological processes	Stable predictors (genetic); Integrates lifetime risk; Potentially highly specific	Typically not modifiable; Requires specialized testing

**Table 6:** Neuroimaging Methods in Delirium Research. This table outlines the neuroimaging approaches used in delirium research, including specific techniques, key applications, major findings, and practical considerations. The table focuses primarily on human studies, with notes on which techniques can be adapted for animal models. Abbreviations: MRI—magnetic resonance imaging; FLAIR—fluid-attenuated inversion recovery; fMRI—functional MRI; PET—positron emission tomography; FDG—fluorodeoxyglucose; TSPO—translocator protein; EEG—electroencephalography. References: [47,128,129,130,131,132,133,134,135].

Imaging Modality	Specific Techniques	Key Applications in Delirium	Major Findings	Practical Considerations
Structural MRI (Adaptable for rodent models with specialized equipment)	T1/T2-weighted imaging; FLAIR; Diffusion tensor imaging; Volumetric analysis	Identifying preoperative risk factors; Assessing white matter integrity; Detecting atrophy patterns	White matter hyperintensities predict delirium risk; Reduced brain volume (especially hippocampus); Disrupted white matter tract integrity	Widely available; Patient motion limitations; Challenges in acutely ill patients
Functional MRI (Adaptable for rodent models with specialized equipment)	Resting-state connectivity; Task-based activation; Default mode network analysis	Mapping functional connectivity changes; Identifying network disruptions; Correlating activity with symptoms	Disrupted default mode network connectivity; Altered frontoparietal network function; Reduced network integration	Requires patient cooperation; Limited feasibility during active delirium
PET Imaging (Adaptable for rodent models with specialized equipment)	FDG-PET (metabolism); Neuroinflammation tracers; Neurotransmitter system tracers	Measuring cerebral metabolism; Quantifying microglial activation; Assessing neurotransmitter function	Reduced glucose metabolism during delirium; Increased TSPO binding (microglial activation); Altered cholinergic receptor availability	Limited availability; Radiation exposure; Cost and technical complexity
Electroencephalography (EEG) (Adaptable for rodent models)	Quantitative EEG; Event-related potentials; Spectral analysis	Continuous monitoring of brain activity; Early detection of delirium; Severity assessment	Slowing of background rhythm; Reduced alpha and increased delta power; Altered functional connectivity	Bedside application feasible; Continuous monitoring possible; Non-invasive

**Table 7:** Therapeutic Targets for Postoperative Delirium. This table outlines the therapeutic targets for postoperative delirium, with information on mechanisms of action, intervention examples, preclinical evidence (primarily from rodent models), and clinical development status. Abbreviations: IL—interleukin; TNF—tumor necrosis factor; BBB—blood–brain barrier; nAChR—nicotinic acetylcholine receptor; NMDA—N-methyl-D-aspartate; ROS—reactive oxygen species; ATP—adenosine triphosphate; MMP—matrix metalloproteinase; SPMs—specialized pro-resolving mediators. References: [157,170,171,172,173,174,175,176,177,178,179].

Target Category	Specific Targets	Mechanism of Action	Examples of Interventions	Preclinical Evidence	Clinical Development Status
Neuroinflammatory Pathway Targets	IL-1 receptor; TNF-α signaling; Microglial activation; Inflammasome components; Leukocyte recruitment	Reduction in pro-inflammatory cytokines; Attenuation of microglial activation; Prevention of BBB disruption; Enhancement of inflammatory resolution	IL-1 receptor antagonists; TNF-α inhibitors; Specialized pro-resolving mediators; Microglial modulators (minocycline); Chemokine receptor antagonists	Prevention of cognitive deficits in aged mice models; Reduced neuroinflammation; Preserved neuronal function	Phase II trials for IL-1Ra; Repurposed biologics in pilot studies; SPMs in preclinical-to-clinical transition
Neurotransmitter System Targets	Cholinergic receptors; Acetylcholinesterase; Dopamine D2/D3 receptors; NMDA receptors	Enhancement of cholinergic function; Modulation of dopaminergic signaling; Optimization of excitatory/inhibitory balance; Restoration of neurotransmitter homeostasis	Cholinesterase inhibitors; α7 nAChR positive allosteric modulators; Atypical antipsychotics; NMDA modulators	Improved attention and memory in aged rodent models; Prevention of neurotransmitter imbalance; Reduction in delirium-like behaviors	Several Phase II/III trials completed; Mixed results for cholinesterase inhibitors; Newer receptor-selective agents in early trials
Metabolic and Energetic Targets	Mitochondrial function; Oxidative stress pathways; Glucose metabolism; Insulin signaling	Protection of mitochondrial integrity; Reduction in ROS damage; Enhancement of ATP production; Support of neuronal energetics	Mitochondria-targeted antioxidants; Electron transport chain modulators; Ketone body supplementation; Metabolic cofactors	Preserved mitochondrial function in aged rodents; Reduced oxidative damage; Enhanced metabolic resilience	Several agents in Phase I safety studies; Nutritional approaches in clinical trials; Mitochondrial drugs in development
Blood–Brain Barrier Targets	Tight junction proteins; Matrix metalloproteinases; Endothelial adhesion molecules; Vascular endothelial growth factor	Preservation of BBB integrity; Reduction in paracellular permeability; Prevention of inflammatory cell infiltration	MMP inhibitors; Sphingosine-1-phosphate receptor modulators; Angiopoietin-Tie2 pathway modulators; Vascular stabilizing agents	Reduced BBB permeability in aged mouse models; Prevention of neuroinflammatory cascade; Preserved neurovascular coupling	Several agents in preclinical validation; Limited clinical trials to date; Repurposed drugs being considered

## Data Availability

No new data were created or analyzed in this study.
